# The Future of Nanotechnology: Healthcare and Manufacturing

**DOI:** 10.3390/nano16211435

**Published:** 2026-11-06

**Authors:** Jihua Chen

**Affiliations:** Center for Nanophase Materials Sciences, Oak Ridge National Laboratory, 1 Bethel Valley Road, Oak Ridge, TN 37830, USA; chenj1@ornl.gov

Nanotechnology has shifted from demonstrating control over matter at the scale of a few billionths of a meter to building products that should work reliably and safely in clinics, factories, and the environment. The field began as a proposition about control. Feynman’s 1959 lecture imagined arranging matter with a precision far beyond the tools available then [1], and the scanning tunneling microscope later made atomic-scale surface structure experimentally visible [2]. Colloidal synthesis, lithography, thin-film deposition, self-assembly, and printing progressively extended that control to materials and devices, and the 1995 approval of pegylated liposomal doxorubicin (Doxil) marked an early landmark for clinical nanomedicine [3].

The next phase will be defined less by what can be made at the nanoscale than by how consistent nanoscale structure is connected to function, how laboratory recipes lead to reproducible production, and how reliably measurements transition to decisions. Five contributions to this Special Issue, four reviews and one research article illuminate that transition. Spada and Gerber-Lemaire investigate how nanocarriers can be decorated with ligands that recognize the epidermal growth factor receptor (EGFR), a receptor overexpressed in many cancers [4]; Alfei and Zuccari survey applications alongside nanotoxicology and public acceptance [5]; Chen and co-workers synchronize additive manufacturing and electronics printing with artificial intelligence (AI) [6]; Sengupta and Hussain trace two decades of titanium dioxide (TiO_2_) nanotube sensors for medical diagnostics [7]; and Flores-Sáenz and co-workers validate a standardized protocol for administering nanomaterials into the rat knee joint [8]. Read together, they share one premise: designed function must be distinguished from demonstrated function, and laboratory performance from real-world benefit.

Spada and Gerber-Lemaire treat active cancer targeting as a surface-engineering problem [4]. They compare epidermal growth factor itself, peptides (such as GE11), antibodies and their fragments, nanobodies, affibodies, aptamers, and small molecules that recognize the epidermal growth factor receptor, a receptor overexpressed in many cancers, together with the chemistries used to attach them. Because ligand orientation, density, and linkage stability decide whether a targeting element remains functional, the carrier surface becomes part of the therapeutic design rather than a passive boundary around the payload. Measuring how much ligand is present does not show that it is oriented or active, while tumor accumulation, penetration, uptake, trafficking, and release are separate steps; therefore, surface composition needs to be linked to functional assays.

Alfei and Zuccari relate nanoparticles, dendrimers, micelles, and liposomes to drug delivery, bioavailability, and food packaging [5], showing why no single account of nanomaterial benefit or risk is adequate. Exposure may occur during synthesis, use, residence in the body, or disposal; dissolution, aggregation, and aging alter the material being evaluated; toxicity therefore should not be inferred from a pristine particle or a single assay [9]. Development should weigh function against solvent and energy demand, persistence, and recovery from the initial stages and, for medical products, evaluate the attributes that nanomaterials introduce, as described in U.S. Food and Drug Administration (FDA) guidance [10]. Multi-objective models can help organize this evidence, but measurements, dose definitions, and uncertainty must remain visible. Responsible development is most effective when it shapes designs while alternatives are still available.

Chen and co-workers show how functional materials and three-dimensional manufacturing can be designed together for energy storage, sensors, wearables, organic electrochemical transistors, functional and intelligent soft systems [6]. The performance of a nanomaterial ink depends on dispersion, particle shape, flow behavior, deposition, and curing; printed geometry then shapes device properties. AI can support defect detection, models relating process settings to outcomes, as well as complex optimization across competing objectives such as conductivity, resolution, flexibility, and cost; digital twins—continuously updated computational replicas of a process—are useful when they are validated against measurements. Because adapting a process during production changes the conditions under which quality was specified, a learning process demands a defined range of permitted adjustments, checks that identify excursions, and records linking every item or batch to its inputs and history. Co-designing ink chemistry, device architecture, deposition, and inspection could benefit patient-fitted wearables and soft bioelectronics.

Sengupta and Hussain trace TiO_2_ nanotubes from controllable nanostructures to sensing interfaces [7]. High surface area, tunable morphology, chemical stability, and electrochemical or photoelectrochemical activity support detection of metabolic, disease-associated, and infectious targets using enzymes, antibodies, aptamers, or polymers. The still-present gap to a useful diagnostic lies in fabrication reproducibility, long-term stability, selectivity in complex fluids (such as blood), portable readout, as well as clinical validation. Sample collection, surface fouling, calibration, interference, and loss of bioreceptor activity can determine the result in practice, so the relevant comparison is a complete measurement procedure in its intended setting rather than a value in buffer. Continuous or wearable sensing raises questions of maintenance, missing data, and drifting baselines. Sensors should therefore be co-developed with sampling, reference channels, packaging, and interpretation, and judged on whether they improve clinical decisions and outcomes.

Flores-Sáenz and co-workers address an important step that is often regarded as routine: delivering a nanomaterial to its intended site [8]. Their protocol for intra-articular injection into the rat knee specifies the anatomical landmark. Histology confirmed intra-articular localization of the injected particles. Verifying where a dose arrives, rather than assuming it, strengthens reproducibility and supports the refinement of animal research.

To summarize, in the Special Issue, there are three threads that run across these contributions.

The first is measurement. A ligand count, a sensitivity in buffer, a printed device’s conductivity, and an injected volume are inputs; the outcomes that matter are target engagement, a correct clinical decision, a device that works across batches, and a dose delivered where it was intended. Orthogonal characterization, complete reporting of procedures, and records that are findable, accessible, interoperable, and reusable (FAIR) [11] allow results from one laboratory to be reused in another.

The second is nanoscience with defined boundaries. The design spaces described here, from ligand density and carrier size to ink formulation and print settings, suit structured experiments, active learning, and closed-loop laboratories [12]. Their value depends on objectives that reflect real benefit, on checks that keep adaptation within validated conditions, and on qualified human authorization for hazardous or clinically consequential steps.

The third is the balance between efficiency and ethics. The replacement, reduction, and refinement of animal use, the three Rs first articulated by Russell and Burch [13], now have regulatory momentum: the FDA Modernization Act 2.0 removed the statutory requirement that drugs be tested in animals before human trials [14], and the FDA’s 2025 roadmap describes a staged reduction in animal testing through validated alternatives [15]. Organ-on-chip devices and other human-relevant models [16] can address many of the issues posed earlier. Open software, data, and hardware can broaden who contributes to all of this [17,18], provided their outputs are validated.

For the upcoming nanotechnologies in healthcare and manufacturing, the priorities are proposed as follows: (1) Characterize materials under the conditions that determine their function, using orthogonal and calibrated methods; (2) publish reproducible protocols, code, and informative failures in FAIR form; (3) assess the lifecycle, environmental, safety, and maintenance burdens early, while designs can still change, using AI screening and human-relevant models to minimize animal testing; and (4) validate diagnostic, therapeutic, and manufacturing systems across sites repeatedly.
